# Patient perceptions of the re-usable Respimat^®^ Soft Mist™ inhaler in current users and those switching to the device: A real-world, non-interventional COPD study

**DOI:** 10.1177/1479973120986228

**Published:** 2021-02-01

**Authors:** Michael Dreher, David Price, Asparuh Gardev, Pascale Peeters, Satish Arora, Simone van der Sar – van der Brugge, Richard Dekhuijzen, Omar S Usmani

**Affiliations:** 1 Department of Pneumology and Intensive Care Medicine, 39058 University Hospital Aachen; RWTH Aachen University, Aachen, Germany; 2 Observational and Pragmatic Research Institute, Singapore; 3 Optimum Patient Care, Cambridge, UK; 4 Centre of Academic Primary Care, Division of Applied Health Sciences, University of Aberdeen, Aberdeen, UK; 5 60325 Boehringer Ingelheim International GmbH, Ingelheim am Rhein, Germany; 6 54886 IQVIA RDS France, La Défense, France; 7 Lambertseter Medical Centre, Oslo, Norway; 8 89411 Amphia Hospital, Breda, The Netherlands; 9 6029 Radboud University Medical Center, Nijmegen, The Netherlands; 10 National Heart and Lung Institute (NHLI), 4615 Imperial College London, London, UK

**Keywords:** COPD, Respimat, switch, preference, PASAPQ, ease of handling

## Abstract

The Respimat^®^ Soft Mist™ inhaler (SMI) has recently been improved, with a re-usable device replacing the disposable version. Certain countries are currently phasing out the disposable inhaler. This study aimed to assess patient satisfaction with and preference for the re-usable device. This 4–6-week, multicentre, open-label, prospective, real-world, non-interventional study was conducted across six European countries. Patients with chronic obstructive pulmonary disease were enrolled between October and December 2019, in three cohorts: (1) currently using the re-usable Respimat SMI; (2) switched from disposable Respimat SMI at study entry; and (3) naïve to any Respimat SMI. Patients were assessed using the Patient Satisfaction and Preference Questionnaire (PASAPQ) and Ease of Handling Questionnaire. In total, 262 patients were enrolled. At follow-up, the mean PASAPQ score was 83.3/100 overall, with similar results across all three patient cohorts. Most patients were ‘satisfied’ or ‘very satisfied’ with the re-usable device. The overall score for willingness to continue using the device was 87.8/100. In total, 13 adverse events were recorded, none of which was classified as serious. This study provides real-world evidence for practitioners to start patients on Respimat re-usable, irrespective of a patient’s prior experience with this inhaler.

**Plain language summary:**

Inhalers are often used to treat patients with chronic obstructive pulmonary disease (COPD). However, there are many available, which can lead to confusion and poor inhaler technique. It is important for a patient to be happy with their inhaler. This study looked at how patients liked the re-usable Respimat® Soft Mist™ inhaler vs. their previous inhaler. It also asked whether they would be willing to continue using the device at the end of the study period.After 4–6 weeks of using the re-usable device, patients reported that they were happy with the inhaler and most would be willing to carry on using it.Overall, these results show that doctors can prescribe Respimat re-usable to patients, even if the patient has not used the inhaler before.

**Figure:**
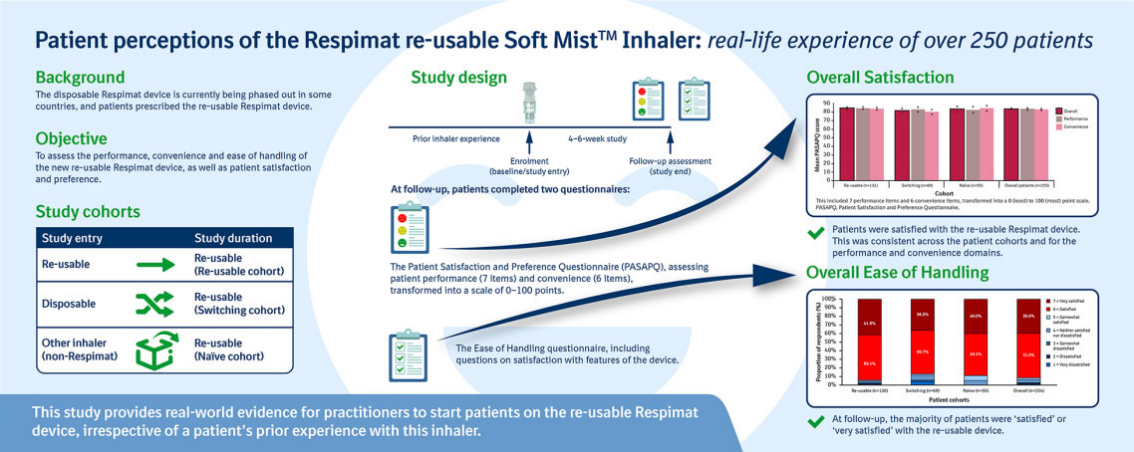


## Introduction

The Respimat^®^ Soft Mist™ inhaler (SMI), first available for patients with chronic obstructive pulmonary disease (COPD) in 2004,^1,2^ was developed to overcome limitations associated with earlier devices such as pressurised metered-dose inhalers (pMDIs), dry powder inhalers (DPIs) and nebulisers.^3,4^ Objectives at the time included avoiding propellant use and minimising the need for patient coordination and inspiratory effort, while optimising drug delivery to the lungs.^3,4^ The first Respimat device was disposable, locking after the labelled number of doses was reached, thereby requiring a new inhaler each month.^4^ This overcame the problem of patients continuing to use their inhaler when empty, which was commonly reported with some DPIs and pMDIs.^5–8^ Since then, development efforts have focused on improving use of the Respimat SMI from the patient’s perspective and allowing the inhaler to be re-usable, while maintaining drug delivery to the lungs.^3^

Respimat re-usable,^1,3^ available for use in Europe since 2019, represents an evolution of the original disposable device following patient/physician feedback.^1^ The new device has improved usability (design changes have enabled use with up to six cartridges), a clearer dose indicator on each cartridge, a reversible lock to allow for re-usability, along with a memory aid to monitor the number of cartridges used.^1^ Studies demonstrating the benefits of these design modifications have previously been published.^1,9,10^

Starting in March 2019, many countries across Europe began phasing out the disposable device in favour of the re-usable device. Given this trend, it is important to assess the effect of switching device on patient satisfaction and preference in real-world settings.

This study aimed to assess the performance, convenience and ease of handling of the re-usable Respimat SMI in routine clinical practice, as well as patient satisfaction and preference.

## Methods

### Study design

**Figure 1.**
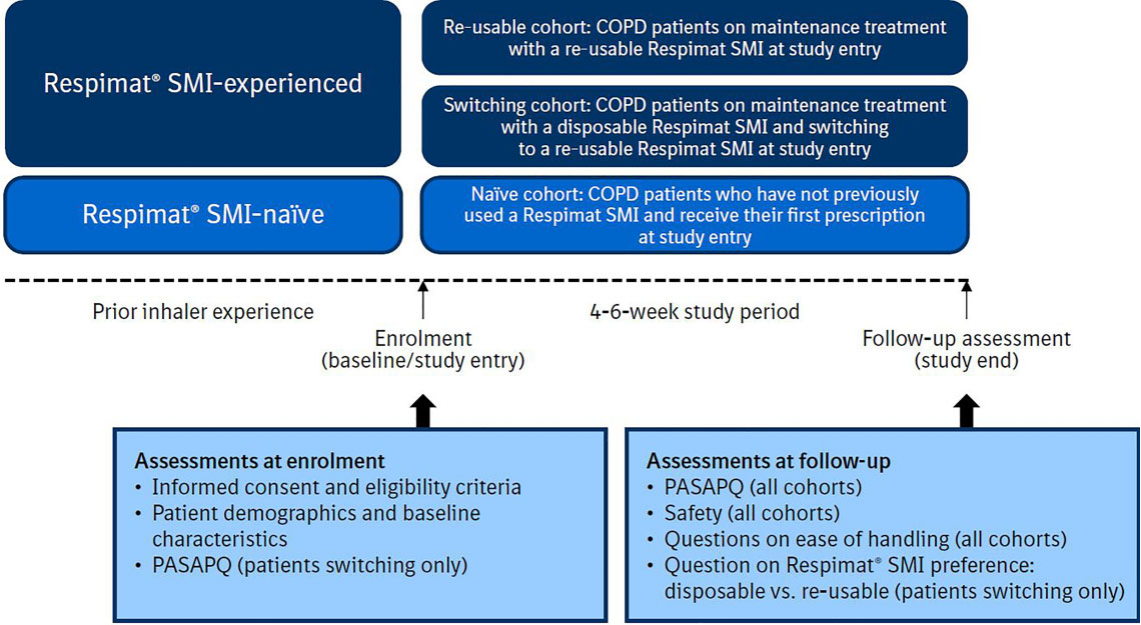
Study design. COPD, chronic obstructive pulmonary disease; PASAPQ, Patient Satisfaction and Preference Questionnaire; SMI, Soft Mist™ inhaler.

This multicentre, open-label, prospective, real-world, non-interventional study (NIS) was conducted at 20 sites across six European countries (Belgium, Denmark, Finland, Germany, the Netherlands and Norway). The study included patients with COPD who were prescribed a re-usable Respimat SMI product. It included three cohorts: (1) patients already receiving maintenance treatment with the re-usable SMI (‘Re-usable’ cohort); (2) patients switching from the disposable to the re-usable SMI (‘Switching’ cohort); and (3) patients who had not previously used any SMI (‘Naïve’ cohort) (Figure 1).

Patients in the three cohorts were prescribed the re-usable SMI, as per routine clinical practice at the participating sites. Prescribing physicians received no guidance regarding training of the participants on how to use the re-usable device. Patients were permitted to use other inhalers for maintenance treatment in addition to the Respimat device; these were recorded and exploratory analyses conducted based on this information.

Patients were enrolled between October and December 2019 by participating physicians involved in the diagnosis, treatment and management of COPD, including general practitioners and secondary care specialists. Each patient was followed prospectively from the time of enrolment until the end of the study period (4–6 weeks after enrolment), loss to follow-up or death. The last patient was assessed in February 2020.

### Study population

Eligible patients provided written informed consent prior to inclusion in the study. Each country provided study approval from a local ethics committee or a central ethics committee (see Supplemental material). This study is registered on ENCePP (EUPAS30293). Patients were enrolled if they had a diagnosis of COPD, were aged 40 years or older, were newly prescribed or currently receiving Respimat SMI products (Spiriva^®^ 2.5 µg, Striverdi^®^ 2.5 µg or Spiolto^®^ 2.5 µg/2.5 µg inhalation solution) and were literate in one of the main languages of their country. In the prescribing physician’s opinion, patients were unlikely to change therapy during the observation period.

Patients were excluded if they used a disposable Respimat SMI after study entry, experienced a severe COPD exacerbation requiring hospitalisation in the 3 months prior to study entry, were currently participating in another clinical trial or NIS, or if they had an impairment that raised concerns regarding their ability to complete the questionnaires.

### Study outcomes and assessments

The Patient Satisfaction and Preference Questionnaire (PASAPQ), validated in patients with asthma and COPD,^11^ was used to assess patient responses. The PASAPQ is a self-administered, multi-item instrument that includes a performance domain (seven items: question [Q] 1–5, Q10–11), a convenience domain (six items: Q6–9, Q12–13), and standalone questions on overall satisfaction (Q14) and willingness to continue with an inhaler (Q15) (see Supplemental material).^11^

Each PASAPQ item has Likert-type response options ranging from 1 to 7 (1 = very dissatisfied to 7 = very satisfied). The PASAPQ total score is the sum of the 13 performance and convenience domain items, transformed into a 0–100 (least–most) point scale, with higher scores indicating greater satisfaction. To calculate a domain score, the patient must have answered at least half of the domain items, with the total score calculated when both the performance and convenience domains had computable scores.

For Q14 (overall satisfaction), scoring ranged from 1 to 7 (very dissatisfied to very satisfied), and for Q15 (willingness to continue using the device), scoring ranged from 0 (not willing) to 100 (definitely willing), with values ≥60 considered to represent willingness to continue.

The PASAPQ was administered at study entry for the ‘Switching’ cohort and at follow-up for all cohorts (Figure 1). The minimum important difference (MID) was defined as 8–10 points.^11^

The Ease of Handling questionnaire was administered at follow-up for all patients. This questionnaire, developed by Boehringer Ingelheim (see Supplemental material), is based on close observations from the Usability Tests of Respimat re-usable,^1^ and measures patient satisfaction with 10 attributes of the device on a seven-point Likert scale similar to the PASAPQ.

Supplementary question 1 of the Ease of Handling questionnaire determined preference for the type of Respimat SMI.

All patients were asked to report any adverse events (AEs) at follow-up; an investigator then assessed whether these could be classified as serious adverse drug reactions (ADRs).

### Statistical analysis

All analyses were conducted in the overall population and in each of the cohorts, except for device preference, which was assessed in the switching cohort only. The primary outcome was the mean total PASAPQ score at study end; secondary outcomes included scores for the PASAPQ performance and convenience sub-domains. In the switching cohort, differences in the mean PASAPQ total score, and performance and convenience scores, were assessed at study entry and follow-up. Mean PASAPQ scores were also analysed according to the number and type of maintenance inhalers used during the study.

The full analysis set (FAS), comprising all enrolled subjects who met the eligibility criteria and who received at least one dose using the re-usable Respimat SMI, was used for all primary and secondary outcome analyses.

All statistical tests were two-sided, assessed using a significance level of 0.05; these were exploratory and not adjusted for multiplicity. PASAPQ scores were summarised using descriptive statistics for continuous variables; overall satisfaction was summarised using descriptive statistics for categorical variables. Ease of handling attributes are presented as categorical variables. Statistical analyses for the switching population included Wilcoxon signed-rank tests.

## Results

### Patient cohort

**Figure 2.**
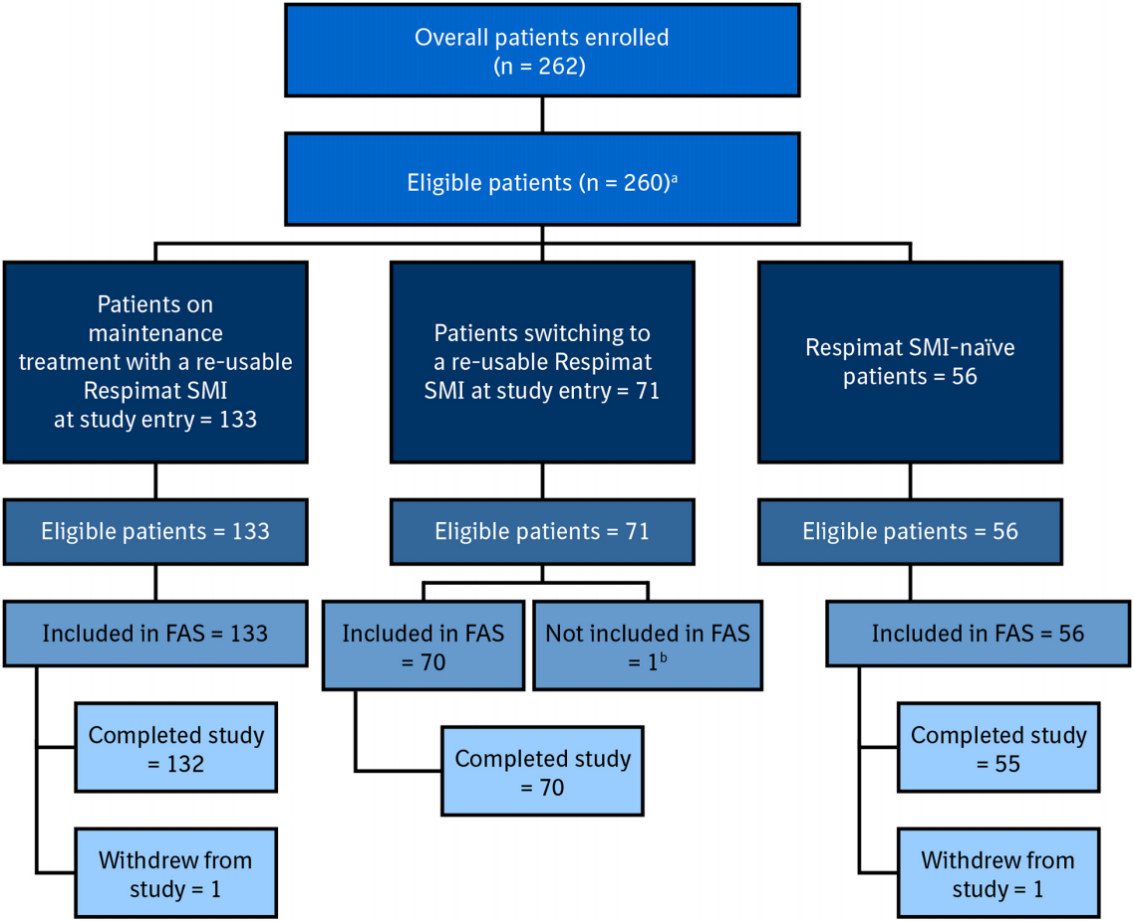
Patient disposition. ^a^One patient did not meet the inclusion criteria, and one patient met the exclusion criteria. ^b^Patient received disposable inhaler instead of Respimat re-usable. FAS, full analysis set; SMI, Soft Mist™ inhaler.

Overall, 262 patients were enrolled, of which 259 were included in the FAS (133 re-usable, 70 switching, 56 naïve). In total, 257 patients completed the study (132 re-usable, 70 switching, 55 naïve) (Figure 2).

Table 1 shows the patient demographics and baseline characteristics of the FAS. Of these, 17.0% were identified from primary care settings, 18.9% were from settings providing both primary and secondary care, and 64.1% were from secondary care facilities. Information regarding previous device and medication use can be found in the Supplemental material.

**Table 1.** Patient demographics and baseline characteristics.

	Re-usable (n = 133)	Switching (n = 70)	Naïve (n = 56)	Overall (n = 259)
**Mean age, years (SD)**	69.1 (7.59)	68.6 (8.58)	68.7 (7.69)	68.9 (7.86)
**Gender, n (%)**				
Male	67 (50.4)	33 (47.1)	31 (55.4)	131 (50.6)
Female	66 (49.6)	37 (52.9)	25 (44.6)	128 (49.4)
**Age at diagnosis, mean years (SD)**	60.4 (9.62)	58.6 (8.96)	61.3 (9.74)	60.1 (9.49)
**Duration of illness, mean years (SD)**	9.1 (5.72)	10.54 (6.83)	7.83 (7.19)	9.21 (6.41)
**Education level, n (%)**				
Elementary school	56 (42.4)	30 (44.1)	32 (58.2)	118 (46.3)
High school	66 (50.0)	34 (50.0)	18 (32.7)	118 (46.3)
University degree	10 (7.6)	4 (5.9)	5 (9.1)	19 (7.5)
Missing	1	2	1	4
**COPD GOLD stage, n (%)**				
1	6 (4.5)	5 (7.1)	6 (10.7)	17 (6.6)
2	28 (21.1)	24 (34.3)	21 (37.5)	73 (28.2)
3	44 (33.1)	20 (28.6)	10 (17.9)	74 (28.6)
4	9 (6.8)	4 (5.7)	2 (3.6)	15 (5.8)
Missing/GOLD assessment not performed	46 (34.6)	17 (24.3)	17 (30.4)	80 (30.9)
**Care facility, n (%)**				
Primary	25 (18.8)	8 (11.4)	11 (19.6)	44 (17.0)
Primary/Secondary	22 (16.5)	19 (27.1)	8 (14.3)	49 (18.9)
Secondary	86 (64.7)	43 (61.4)	37 (66.1)	166 (64.1)

COPD, chronic obstructive pulmonary disease; GOLD, Global Initiative for Chronic Obstructive Lung Disease; SD, standard deviation.

The mean duration of participation for patients who completed the study was 37.2 days (standard deviation 6.6).

### PASAPQ total score, performance and convenience domain scores at study end

Overall, 255 patients completed the PASAPQ (re-usable, n = 131, switching, n = 69; naïve, n = 55). The mean total PASAPQ score was 83.3 out of 100 (95% confidence interval [CI] 81.2–84.6), indicating that patients were satisfied with Respimat re-usable. This was consistent across the three cohorts (means: 81.6–84.3), the performance and convenience domains (means: 82.4–84.7 and 80.1–84.2, respectively) and the item scores individually (see Supplemental material).

In the switching cohort, the mean PASAPQ performance and convenience scores increased from 80.9 and 79.3 at baseline to 82.9 and 80.1 at follow-up, respectively, although these were not statistically significant (Wilcoxon signed-rank test P = 0.409 and P = 0.932, respectively) and did not meet the MID of 8–10 points.^11^ Factors such as instructions for use, ease of cleaning and ease of holding the inhaler scored higher at follow-up than at study entry.

Irrespective of other maintenance devices being used during the study, mean PASAPQ scores and performance and convenience scores were similar: re-usable only, means 82.7–83.5 (n = 144); re-usable + DPI, means 85.1–86.7 (n = 45); re-usable + MDI, means 81.2–81.5 (n = 64); re-usable + MDI + DPI, means 97.2–100 (n = 2) (see Supplemental material).

When analysed by age, median PASAPQ scores were significantly higher in patients aged <65 years compared with those aged ≥65 years (<65 years: 85.9, ≥65 years: 83.3; P = 0.009), though the numerical difference was lower than the MID.

### PASAPQ satisfaction (Q14) and willingness to continue (Q15)

**Figure 3.**
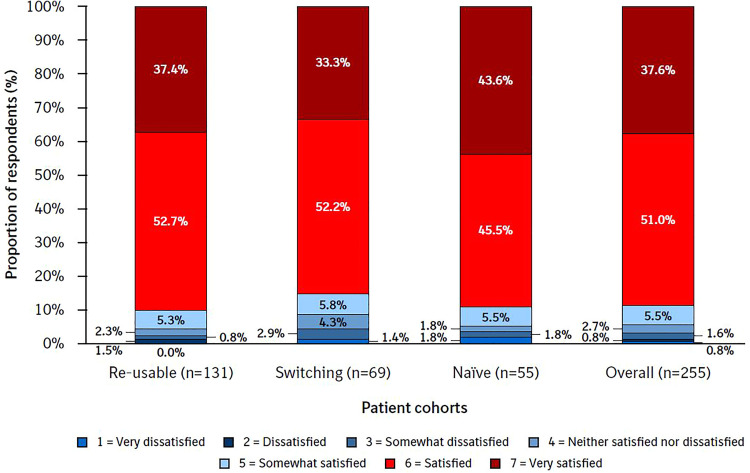
Overall satisfaction by cohort. PASAPQ question 14. The Overall Satisfaction item response values range from 1 (very dissatisfied) to 7 (very satisfied). PASAPQ, Patient Satisfaction and Preference Questionnaire.

The majority of patients (88.6%) were ‘satisfied’ (51.0%) or ‘very satisfied’ (37.6%) with Respimat re-usable (Figure 3). Overall, 3.2% of patients reported that they were very dissatisfied, dissatisfied or somewhat dissatisfied with the re-usable inhaler (range: 4.3% in the switching cohort to 2.3% in the re-usable cohort).

Regarding willingness to continue using the re-usable SMI, the overall mean score was 87.8 (95% CI 85.7–89.8), indicating that the majority of patients would be willing to continue using the inhaler (this was similar across all patient cohorts [means 84.1–89.1]; see Supplemental material).

### Ease of Handling questionnaire

**Figure 4.**
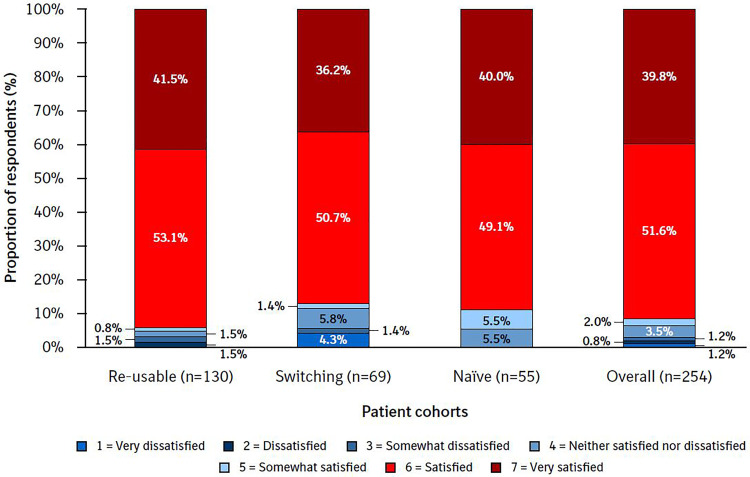
Overall ease of handling of Respimat re-usable. Ease of Handling questionnaire (Q8). The Ease of Handling item response values range from 1 (very dissatisfied) to 7 (very satisfied).

Overall, 255 participants completed the Ease of Handling questionnaire (re-usable, n = 131, switching, n = 69; naïve, n = 55). Of these, 254 responded to Q8 on ‘overall ease of handling’: 91.3% of patients responded that they were ‘satisfied’ (score = 6) or ‘very satisfied’ (score = 7) (re-usable 94.6%, switching 87.0%, naïve 89.1%) (Figure 4), and 3.1% of patients responded that they were very dissatisfied (score = 1), dissatisfied (score = 2) or somewhat dissatisfied (score = 3) (range: 5.8% in the switching cohort to 0.0% in the naïve cohort).

For the other questions in the Ease of Handling questionnaire, 89.8% of patients gave scores of 6 or 7 for recognising when they needed to replace the cartridge (Q5), and 89.3% for the readability of the dose indicator (Q4) and grip of the cartridge (Q2). For the remaining questions, the percentage of patients who gave scores of 6 or 7 ranged from 89.0% (for recognising when to replace the inhaler [Q10]) to 77.2% (satisfaction with inserting a new cartridge [Q3]) (see Supplemental material).

### Patient preference (supplementary question 1)

In the switching cohort, 68 of the 70 patients responded to the inhaler preference question. Of the respondents, 57 (84%) preferred Respimat re-usable; eight (12%) preferred the disposable device and three (4%) indicated no preference.

Of the eight patients who reported a preference for the disposable Respimat, five were enrolled from the same site. The majority of the eight patients gave PASAPQ scores >4 for the performance and convenience items, indicating some level of satisfaction with the re-usable device. Regarding Q15 of the PASAPQ, ‘willingness to continue’, six of these eight patients (75%) reported that they would be willing to continue using Respimat re-usable (ratings of 75 [n = 1], 85 [n = 1], and 100 [n = 4], respectively).

Regarding the Ease of Handling questionnaire, scores among the eight patients who preferred the disposable Respimat were lower for the additional handling steps involved with the re-usable device. Specifically, lower scores were reported for the steps relating to cartridge replacement, including ease of removing the clear base (Q1), inserting a new cartridge (Q3), automatic detachment of clear base (Q6) and automatic return to start-use position (Q7) (range: 4.75–4.88), compared with the overall population (range: 5.77–5.94) and with switching patients who preferred the re-usable device (range: 5.66–5.91).

### Safety

In total, 13 AEs were reported, none of which was classified as serious. These included one ADR (sore throat) that resulted in treatment discontinuation in a Respimat-naïve patient.

## Discussion

The present study shows that, in clinical practice, patients report satisfaction with Respimat re-usable in terms of performance, convenience and ease of handling. Consistent levels of satisfaction were reported among patients already using the device, those who switched to the device from the disposable device, and new users, as well as patients using multiple maintenance inhalers.

The current study supports previous data that showed high patient satisfaction with SMIs,^4,8,12,13^ including studies using the PASAPQ and similar questionnaires.^13–15^ Previous reports on satisfaction using the PASAPQ have indicated that a score of 6.0 (or over 80.00 when transformed) indicates high patient satisfaction.^13–15^ The mean PASAPQ total score of 83.3 for the re-usable SMI was slightly higher than that reported in a previous study evaluating the disposable Respimat (80.7).^14^ These differences in scores are within the MID.^11^

In the convenience domain, the switching cohort reported slightly lower scores than other cohorts. As these patients were required to switch from their disposable Respimat inhalers, their perception of convenience may understandably be lower than in patients who did not switch at study entry or who were naïve to the device. Despite this, their convenience score was still high, with factors such as instructions for use, ease of cleaning and ease of holding the inhaler scoring higher at follow-up than with their previous device. Responses to the PASAPQ question about ‘using the inhaler’ also improved at follow-up, in line with follow-up results from the Ease of Handling questionnaire. Therefore, switching from the disposable to the re-usable device appears to be associated with high levels of satisfaction overall.

Regarding willingness to continue using Respimat re-usable after study completion, the results were similar across all three cohorts, with patients already on the re-usable device having the highest mean score (89.1) and Respimat-naïve patients having a slightly lower score (84.1). This may be due to their lack of experience with the Respimat inhaler. The naïve cohort scored highest overall on device convenience (84.2), followed by the re-usable cohort (83.7) and then the switching cohort (80.1).

Patient satisfaction and correct inhaler use are critically important for optimal drug delivery and can predict favourable clinical outcomes.^1,16,17^ Ineffective training on an inhalation device can influence the patient’s perception of the device performance;^18,19^ however, as this was an NIS, reflecting general clinical practice, no guidance was given to the physicians regarding training of the participants on the re-usable device. The majority of the switching cohort (84%) reported preference for Respimat re-usable at study end, with 12% preferring the disposable device and 4% reporting no preference. Of the 12% who preferred the disposable device, 75% reported willingness to continue using the re-usable device.

Respimat re-usable was developed in response to feedback from patients.^1,3^ In the current study, patients were most satisfied with the readability of the amount of medication left, ease of inhaling and ease of use, as well as ease of holding the device. Patients also scored steps around cartridge replacement highly (including ease of removing the clear base, inserting a new cartridge, automatic detachment of the clear base and automatic return to start-use position), but scored lower for other questions from the Ease of Handling questionnaire. As such, these may be potential areas to highlight during patient training at the time of inhaler initiation.

This study has a number of strengths, including the use of a validated tool (PASAPQ) to assess patient satisfaction. Furthermore, the large, multinational study population is reflective of a real-world population of patients with COPD (mean age 69.0 years in the current study and 70.8 years in a study by Halpin et al.),^20^ encompassing diversity in terms of inhaler use (including type and multiplicity of inhalers). The study included patients across a range of care settings and Global Initiative for Chronic Obstructive Lung Disease stages.

This study does have some limitations, such as the relatively small sub-groups. Additionally, due to the short study duration, it is possible that all study participants could not assess all aspects of device functionality. However, the mean study duration (37.2 days) implies that a high proportion of participants completed more than 30 days of treatment, and therefore had to replace the cartridge (see Q3 and Q5 of the Ease of Handling questionnaire).

This study suggests that patients preferred the re-usable device to their previous devices; however, the important factor for consideration of medication delivery to the lungs – inhaler technique – was not assessed, with items 1 and 2 of the PASAPQ only asking about the patient’s feeling and confidence that the inhaled medication went to their lungs. Lastly, as this was an open-label, non-randomised study, there is the potential for selection bias, which may create imbalances when comparing between the cohorts. Information on cognitive function, psychological status and patients’ level of social support was not collected, and as such we were unable to assess the impact of these factors on the patients’ ability to correctly use their inhalation devices.

## Conclusions

Patients in this study reported high satisfaction with the performance, convenience and ease of handling of Respimat re-usable. This study provides real-world evidence that can give practitioners confidence to start patients on Respimat re-usable, irrespective of a patient’s prior experience with the Respimat inhaler.

## Supplemental material

Supplemental Material, sj-pdf-1-crd-10.1177_1479973120986228 - Patient perceptions of the re-usable Respimat^®^ Soft Mist™ inhaler in current users and those switching to the device: A real-world, non-interventional COPD study Click here for additional data file. Supplemental Material, sj-pdf-1-crd-10.1177_1479973120986228 for Patient perceptions of the re-usable Respimat^®^ Soft Mist™ inhaler in current users and those switching to the device: A real-world, non-interventional COPD study by Michael Dreher, David Price, Asparuh Gardev, Pascale Peeters, Satish Arora, Simone van der Sar – van der Brugge, Richard Dekhuijzen and Omar S Usmani in Chronic Respiratory Disease
