# Supplementary material for: Patient perceptions of the re-usable Respimat® Soft Mist™ inhaler in current users and those switching to the device: A real-world, non-interventional COPD study
Source: Chron Respir Dis. 2021 Feb 1;18:1479973120986228. doi: 10.1177/1479973120986228 (PMC7868496; doi:10.1177/1479973120986228)
Supplement: Supplemental Material, sj-pdf-1-crd-10.1177_1479973120986228 - Patient perceptions of the re-usable Respimat® Soft Mist™ inhaler in current users and those switching to the device: A real-world, non-interventional COPD study [file sj-pdf-1-crd-10.1177_1479973120986228.pdf]

Supplemental material

Contents

Supplementary Table 1: Ethics approvals 2

Supplementary Table 2: PASAPQ questions and scoring 4

Supplementary Table 3: Ease of Handling questionnaire 5

Supplementary Table 4: Device use prior to study entry and during the study 6

Supplementary Table 5: Ease of Handling summary 8

Supplementary figure legends 9

References 10

Supplementary Table 1: Ethics approvals

Site #	Site country	Ethics type	Authority name	PI name	Account name	Date of submission	Date of approval
101	Belgium	Central Ethics Committee	UZ Brussel	Meysman, Marc	Universitair Ziekenhuis Brussel	10-Sep-19	6-Dec-19
102	Belgium	Central Ethics Committee	UZ Brussel	Baldassarre, Sandra	Clinique Nôtre-Dame de Grâce	10-Sep-19	6-Dec-19
103	Belgium	Central Ethics Committee	UZ Brussel	Perez Bogerd, Silvia	Cliniques Universitaires de Bruxelles Hopital Erasme	10-Sep-19	6-Dec-19
104	Belgium	Central Ethics Committee	UZ Brussel	Carron, Kris	AZ Delta	10-Sep-19	6-Dec-19
201	Denmark	Not applicable	Not applicable	Ulrik, Charlotte	Amager-Hvidovre Hospital	Not applicable	Not applicable
202	Denmark	Not applicable	Not applicable	Hilberg, Ole	Sygehus Lillebælt - Vejle	Not applicable	Not applicable
301	Finland	Central Ethics Committee	Varsinais-Suomen sairaanhotopiiri Eettinen toimikunta	Kilpeläinen, Maritta	Turun yliopistollinen keskussairaala	29-Aug-19	14-Nov-19
302	Finland	Central Ethics Committee	Varsinais-Suomen sairaanhotopiiri Eettinen toimikunta	Ekroos, Heikki	Porvoon sairaala	29-Aug-19	14-Nov-19
303	Finland	Central Ethics Committee	Varsinais-Suomen sairaanhotopiiri Eettinen toimikunta	Kauppinen, Ritva	Etelä-Karjalan keskussairaala	29-Aug-19	14-Nov-19
401	Germany	Central Ethics Committee	Ethik-Kommission an der Medizinischen Fakultaet der Rheinisch-Westfaelischen Technischen Hochschule	Dreher, Michael	Universitaetsklinikum Aachen AOeR	17-Jul-19	26-Sep-19
402	Germany	Local Ethics Committee	Landesärztekammer Rheinland-Pfalz	Schmidt, Olaf	Pneumologische Gemeinschaftspraxis Schaeben, Schmidt, Lobo-Becker, Apine	10-Oct-19	28-Nov-19
404	Germany	Local Ethics Committee	Ethikkommission Schleswig-Holstein	Eberhardt, Frank	Pneumopraxis	11-Oct-19	17-Oct-19
405	Germany	Central Ethics Committee	Ethik-Kommission an der Medizinischen Fakultaet der Rheinisch-Westfaelischen Technischen Hochschule	Schultz, Thomas	MECS GmbH	Not applicable	26-Sep-19
406	Germany	Local Ethics Committee	Ethikkommission bei dar Sächsischen Landesärztekammer	Deckelmann, Regina	Pneumologenzentrum Dr. Deckelmann/ Dr. Eckhardt Dr. Kratzsch	18-Nov-19	4-Dec-19
501	Netherlands	Central Ethics Committee	The Dutch Clinical Research Foundation	Goosens, Martijn	Gelre Ziekenhuizen, Zutphen	12-Jul-19	27-Aug-19
501	Netherlands	Local Ethics Committee	Gelre ziekenhuizen	Goosens, Martijn	Gelre Ziekenhuizen, Zutphen	12-Sep-19	3-Oct-19
502	Netherlands	Local Ethics Committee	BoD Franciscus Gasthuis & Vlietland	In 't Veen, Johannes	Franciscus Gasthuis	11-Sep-19	21-Oct-19
503	Netherlands	Local Ethics Committee	METC Zuyderland	Custers, Frank	Zuyderland Medisch Centrum - Heerlen	5-Nov-19	2-Dec-19

Site #	Site country	Ethics type	Authority name	PI name	Account name	Date of submission	Date of approval
504	Netherlands	Local Ethics Committee	BoD Amphia	van der Sar, Simone	Amphia Ziekenhuis, Molengracht	12-Sep-19	26-Sep-19
505	Netherlands	Local Ethics Committee	Medisch Centrum Leeuwarden	Kuijvenhoven-Varkevisser, Jolanda	Medisch Centrum Leeuwarden	1-Nov-19	13-Nov-19
601	Norway	Central Ethics Committee	Regionale Komiteer For Medisinsk Og Helsefaglig Forskningsetikk	Tomala, Tadeusz	Svelvik Legesenter	22-Sep-19	20-Nov-19
602	Norway	Central Ethics Committee	Regionale Komiteer For Medisinsk Og Helsefaglig Forskningsetikk	Høivik, Hans Olav	Elverum Medisinske Senter AS	22-Sep-19	20-Nov-19
603	Norway	Central Ethics Committee	Regionale Komiteer For Medisinsk Og Helsefaglig Forskningsetikk	Ahlqvist, Jørn	Hisøy legesenter-Kolbjørnsvik	22-Sep-19	20-Nov-19
605	Norway	Central Ethics Committee	Regionale Komiteer For Medisinsk Og Helsefaglig Forskningsetikk	Arora, Satish	Lambertseter medical center	22-Sep-19	20-Nov-19
701	Romania	Central Ethics Committee	Comisia Naţională de Bioetică a Medicamentului	Cardos-Rosca, Loredana Elena	Cardiomed SRL	3-Oct-19	24-Oct-19
703	Romania	Central Ethics Committee	Comisia Naţională de Bioetică a Medicamentului	Ciobotaru, Gabriela-Valentina	S.C Medicali's S.R.L	3-Oct-19	24-Oct-19
704	Romania	Central Ethics Committee	Comisia Naţională de Bioetică a Medicamentului	Olar, Emilia Mariana	Cabinet Medical Individual Dr. Olar Emilia Mariana	3-Oct-19	24-Oct-19
705	Romania	Central Ethics Committee	Comisia Naţională de Bioetică a Medicamentului	Andrei, Laurentia	S.C Centrul Medical de Diagnostic si Tratament Ambulator Neomed S.R.L	3-Oct-19	24-Oct-19

PI, principal investigator.

Supplementary Table 2: PASAPQ questions and scoring

<table>
<tr><th colspan="2">Domain</th><th>Question</th><th>Description</th><th>Scoring</th></tr>
<tr><td rowspan="13">Total score</td><td rowspan="7">Performance domain</td><td>Q1</td><td>Overall feeling of inhaling</td><td rowspan="14">All items scored on a 7-point Likert scale:
1 = Very dissatisfied
2 = Dissatisfied
3 = Somewhat dissatisfied
4 = Neither satisfied nor dissatisfied
5 = Somewhat satisfied
6 = Satisfied
7 = Very satisfied</td></tr>
<tr><td>Q2</td><td>Inhaled dose goes to lungs</td></tr>
<tr><td>Q3</td><td>Amount of medication left</td></tr>
<tr><td>Q4</td><td>Works reliably</td></tr>
<tr><td>Q5</td><td>Ease of inhaling a dose</td></tr>
<tr><td>Q10</td><td>Using the inhaler</td></tr>
<tr><td>Q11</td><td>Speed medicine comes out</td></tr>
<tr><td rowspan="6">Convenience domain</td><td>Q6</td><td>Instructions for use</td></tr>
<tr><td>Q7</td><td>Size of inhaler</td></tr>
<tr><td>Q8</td><td>Durability of inhaler</td></tr>
<tr><td>Q9</td><td>Ease of cleaning inhaler</td></tr>
<tr><td>Q12</td><td>Ease of holding during use</td></tr>
<tr><td>Q13</td><td>Convenience of carrying</td></tr>
<tr><td colspan="2" rowspan="2">Standalone questions</td><td>Q14</td><td>Overall satisfaction</td></tr>
<tr><td>Q15</td><td>Willingness to continue</td><td>Each inhaler given a score between 0 and 100</td></tr>
</table>

Table adapted from Hodder and Price 2009.[1]

PASAPQ, Patient Satisfaction and Preference Questionnaire.

Supplementary Table 3: Ease of Handling questionnaire

Question	Description	Scoring
Q1	How satisfied are you with the ease of removing the clear base?	All items scored on a 7-point Likert scale:<br>1 = Very dissatisfied<br>2 = Dissatisfied<br>3 = Somewhat dissatisfied<br>4 = Neither satisfied nor dissatisfied<br>5 = Somewhat satisfied<br>6 = Satisfied<br>7 = Very satisfied
Q2	How satisfied are you with the grip of the cartridge?	
Q3	How satisfied are you with inserting a new cartridge?	
Q4	How satisfied are you with the readability of the dose indicator?	
Q5	How satisfied are you with recognising when you need to replace the cartridge?	
Q6	How satisfied are you with automatic detachment of the clear base when the cartridge is empty?	
Q7	How satisfied are you with automatic return to the start-use position when replacing the clear base?	
Q8	How satisfied are you with the overall ease of handling the inhaler?	
Q9	How satisfied are you with the sustainability (eco-friendly) concept of the inhaler, due to re-usability?	
Q10	How satisfied are you with recognising when to replace the inhaler?	
Supplementary Q1	**Preference:** Comparing the re-usable with the disposable inhaler, please indicate which inhaler do you prefer to use?	• Prefer inhaler 1<br>• Prefer inhaler 2<br>• No preference

Supplementary Table 4: Device use prior to study entry and during the study

Use	Re-usable (n=133)	Switching (n=70)	Naïve (n=56)
Respimat device use prior to study entry mean days (SD)	Overall: 131.2 days (78.67)<br>Respimat re-usable (Spiriva) (n=50): 130.0 days (71.41)<br>Respimat re-usable (Spiolto) (n=83): 133.2 days (90.17)	Overall: 1305.0 days (1246.99)<br>Respimat disposable (Spiriva) (n=48): 532.6 days (1357.97)<br>Respimat disposable (Spiolto) (n=22): 808.3 days (777.89)	N/A
Other devices and treatments used prior to study entry[a]			
DPI, n (%)	27 (20.3)	12 (17.1)	16 (28.6)
LABA, n (%)	7 (5.3)	2 (2.9)	1 (1.8)
LAMA, n (%)	1 (0.8)	0	4 (7.1)
LAMA+LABA, n (%)	0	1 (1.4)	3 (5.4)
ICS+LABA, n (%)	21 (15.8)	9 (12.9)	9 (16.1)
MDI, n (%)	30 (22.6)	24 (34.3)	10 (17.9)
LABA, n (%)	2 (1.5)	2 (2.9)	1 (1.8)
ICS+LABA, n (%)	28 (21.1)	21 (30.0)	6 (10.7)
ICS+LAMA+LABA, n (%)	0	1 (1.4)	3 (5.4)
DPI+MDI, n (%)	1 (0.8)	0	2 (3.6)
LAMA DPI, n (%)	0	0	2 (3.6)
LABA MDI, n (%)	0	0	1 (1.8)
ICS+LABA DPI, n (%)	1 (0.8)	0	0
ICS+LABA MDI, n (%)	1 (0.8)	0	1 (1.8)
None, n (%)	75 (56.4)	34 (48.6)	28 (50.0)
Inhalers used during the trial			
Only re-usable, n (%)	73 (54.9)	34 (48.6)	40 (71.4)
Re-usable+DPI, n (%)	27 (20.3)	11 (15.7)	8 (14.3)
Re-usable+MDI, n (%)	32 (24.1)	25 (35.7)	7 (12.5)
Re-usable+DPI+MDI, n (%)	1 (0.8)	0	1 (1.8)

[a]Some patients were on multiple inhalers prior to study entry.

DPI, dry powder inhaler; ICS, inhaled corticosteroids; LABA, long-acting β_2-agonist; LAMA, long-acting muscarinic antagonist; MDI, metered-dose inhaler; N/A, not applicable; SD, standard deviation.

Supplementary Table 5: Ease of Handling summary

Question	Description	Number of respondents	Percentage of patients scoring 1–3 (%)	Percentage of patients scoring 6 or 7 (%)
Q1	Ease of removing the clear base	254	7.1	82.7
Q2	Grip of the cartridge	253	4.3	89.3
Q3	Inserting a new cartridge	254	10.2	77.2
Q4	Readability of the dose indicator	253	5.5	89.3
Q5	Recognising when you need to replace the cartridge	254	4.3	89.8
Q6	Automatic detachment of the clear base when the cartridge is empty	252	6.0	81.0
Q7	Automatic return to the start-use position when replacing the clear base	252	6.7	82.1
Q8	Overall ease of handling the inhaler	254	3.1	91.3
Q9	Sustainability (eco-friendly) concept of the inhaler, due to re-usability	254	3.5	87.8
Q10	Recognising when to replace the inhaler	243	3.3	89.3

Supplementary figure legends

Supplementary Figure 1: Overall mean (a) PASAPQ total score and (b) PASAPQ performance and convenience domain scores at follow-up. Individual mean (c) performance and (d) convenience domain item scores

PASAPQ scoring is a sum of the items in each of the domains (questions 1–13), transformed into a 0–100 (least–most) point scale. Dots represent 95% confidence intervals.

PASAPQ, Patient Satisfaction and Preference Questionnaire.

Supplementary Figure 2: PASAPQ scores by number of maintenance inhalers used during the study at follow-up

DPI, dry powder inhaler; MDI, metered-dose inhaler; PASAPQ, Patient Satisfaction and Preference Questionnaire; SE, standard error.

Supplementary Figure 3: Willingness to continue Respimat re-usable

Willingness to continue to use Respimat re-usable (0–100 [not willing–very willing]).

References

1. Hodder R and Price D. Patient preferences for inhaler devices in chronic obstructive pulmonary disease: experience with Respimat Soft Mist inhaler. *Int J Chron Obstruct Pulmon Dis*. 2009; 4: 381-90.
